# Proteomic Analysis of Detergent Resistant Membrane Domains during Early Interaction of Macrophages with Rough and Smooth *Brucella melitensis*

**DOI:** 10.1371/journal.pone.0091706

**Published:** 2014-03-18

**Authors:** Sabine A. Lauer, Srinivas Iyer, Timothy Sanchez, Christian V. Forst, Brent Bowden, Kay Carlson, Nammalwar Sriranganathan, Stephen M. Boyle

**Affiliations:** 1 Los Alamos National Laboratory, Bioscience Division, Los Alamos, New Mexico, United States of America; 2 University of Texas, Southwestern Medical Center, Department of Clinical Sciences, Dallas, Texas, United States of America; 3 Virginia Polytechnic and State University, Department of Biomedical Sciences and Pathobiology and Center for Molecular Medicine & Infectious Diseases, Blacksburg, Virginia, United States of America; East Carolina University School of Medicine, United States of America

## Abstract

The plasma membrane contains discrete nanometer-sized domains that are resistant to non-ionic detergents, and which are called detergent resistant membrane domains (DRMDs) or lipid rafts. Exposure of host cells to pathogenic bacteria has been shown to induce the re-distribution of specific host proteins between DRMDs and detergent soluble membranes, which leads to the initiation of cell signaling that enable pathogens to access host cells. DRMDs have been shown to play a role in the invasion of *Brucella* into host macrophages and the formation of replicative phagosomes called *Brucella*-containing vacuoles (BCVs). In this study we sought to characterize changes to the protein expression profiles in DRMDs and to respective cellular pathways and networks of Mono Mac 6 cells in response to the adherence of rough VTRM1 and smooth 16 M *B. melitensis* strains. DRMDs were extracted from Mono Mac 6 cells exposed for 2 minutes at 4°C to *Brucella* (no infection occurs) and from unexposed control cells. Protein expression was determined using the non-gel based quantitative iTRAQ (Isobaric Tags for Relative and Absolute Quantitation) mass spectrometry technique. Using the identified iTRAQ proteins we performed enrichment analyses and probed constructed human biochemical networks for interactions and metabolic reactions. We identified 149 proteins, which either became enriched, depleted or whose amounts did not change in DRMDs upon *Brucella* exposure. Several of these proteins were distinctly enriched or depleted in DRMDs upon exposure to rough and smooth *B. melitensis* strains which results in the differential engagement of cellular pathways and networks immediately upon *Brucella* encounter. For some of the proteins such as myosin 9, small G protein signaling modulator 3, lysine-specific demethylase 5D, erlin-2, and voltage-dependent anion-selective channel protein 2, we observed extreme differential depletion or enrichment in DRMDs. The identified proteins and pathways could provide the basis for novel ways of treating or diagnosing *Brucellosis*.

## Introduction

Brucellosis caused by Gram-negative coccobacilli of the genus *Brucella* is a severe and persistent infection that may lead to chronic disease with low mortality (fatality <5%), if not treated early [1], [2]. Different species of *Brucella* have been identified and named primarily based on their preferred host animal or features of infection. Brucellosis in humans has been called undulant fever, Malta fever, rock fever, Cyprus fever, Gibraltar fever and Mediterranean fever [3]. Currently, of the six terrestrial and three marine species of *Brucella* [4], four of the terrestrial species have severe to moderate pathogenicity to humans: *B. melitensis* (from sheep & goats; most pathogenic and prevalent worldwide); *B. suis* (from pigs; highly pathogenic); *B. abortus* (from cattle; moderately pathogenic); and *B. canis* (from dogs; moderately pathogenic) [5], [6]. Transmission of the disease by inhalation of aerosols is remarkably efficient because a relatively low number of bacteria (10–100) is needed to establish an infection in humans. This threshold was an important factor in the weaponization of *B. suis*, which became the first biological weapon in the US offensive biological weapons program [3], [7].

Flu-like symptoms of Brucellosis are protean, nonspecific and somatic (weakness, fatigue, malaise, body aches, depression, anorexia) [3]. The pre-symptomatic incubation period for *Brucella* infections ranges between 5 and 60 days, but can be even longer. These long and varying incubation periods and the fact that many infections are asymptomatic, make a timely and definitive diagnosis difficult. Currently, there is no vaccine for human use available to protect against Brucellosis. Infections in humans are treated with combinations of antibiotics such as doxycycline/gentamicin, doxycycline/rifampicin, trimethoprim/sulfamethoxazole (Bactrim), or fluoroquinones such as ciprofloxacin. Even with appropriate and timely treatment protocols, there is a significant relapse rate of 10 to 15%. These unsatisfactory diagnostic and therapeutic options call for new methods development allowing for specific early diagnosis and novel effective treatments, even before the development of clinical signs.

In recent years it has been shown that a growing number of disparate pathogens (viruses, bacteria, protozoa, fungi) utilize specialized membrane domains in their interactions with host cells, including pathogen entry, viral budding, and activation of cell signaling pathways that regulate cell responses. These nanometer-sized membrane domains are enriched in sphingolipids, cholesterol and proteins that interact with the sphingolipid chains and cholesterol, which include glycosylphosphatidylinositol (GPI)- linked proteins, membrane proteins such as caveolins and flotilins, stomatins and doubly acylated proteins and are referred to as detergent resistant membrane domains (DRMDs) or lipid rafts. Cholesterol has a stabilizing effect on these domains since the removal, sequestration, or oxidation of cholesterol destabilizes or disrupts them. The tight acyl packing of lipids in these domains makes them resistant to extraction with low concentrations of non-ionic detergents such as 1% Triton X-100 and provides the basis of their isolation.

Recent studies have shown that DRMDs regulate the internalization and intracellular replication of *B. abortus* and *B. suis* into macrophages [8], [9], [10] and mediate class A scavenger receptor-dependent internalization of *B. abortus* into macrophages [11]. In addition, the DRMD-resident lipids cholesterol and ganglioside GM_1_ have been shown to be required for successful infection of mice by *B. abortus* [9], [12].

It is noteworthy that *Brucella* first requires host cell DRMDs for entry but later produces cyclic β-1,2 glucans to down-regulate DRMD function. DRMDs such as those on phagosomal membranes would lose their signaling capacity which consequently prevents the BCV to fusion with lysosomes, thereby allowing *Brucella* to reach an endoplasmic reticulum-derived vacuole permissive for bacterial replication [13], [14], [15], [16], [17], [18], [19], [20]. Cyclic β-1,2-glucans added exogenously do not prevent the maturation of phagosomes containing latex beads and suggests that interaction of cyclic β-1,2-glucans with other factors produced by *Brucella* is required.

In order to cause persistent infection of the reticuloendothelial system, all human pathogenic *Brucella* species require a type IV secretion system (T4SS) known as VirB [21], [22]. A functional T4SS which includes the translocation of secreted effector proteins into the host cell or the vacuolar membrane is activated in macrophages during intracellular infection [23], [24], [25] and required for the maturation of the BCV into ER-derived replicative organelles, thereby promoting *Brucella's* survival and replication [17], [22], [26], [27], [28]. The expression of the T4SS VirB is tightly regulated both *in vitro* and *in vivo* [23], [24], [25], [29] by several molecular systems. Direct interactions with the promoter region of VirB has been demonstrated for integration host factor, a DNA-binding and -bending protein with roles in local DNA structural organization and transcriptional regulation of a wide variety of bacterial genes [24], VjbR the quorum sensing-dependent regulator involved in surface modifications of *Brucella* [30], [31], HutC, the transcriptional repressor of the histidine genes [32], and the two-component regulatory system BvrR/BvrS (TCS BvrRS) that participates in the homeostasis of the outer membrane controlling the structure of the lipopolysaccharide (LPS), the expression of periplasmic and outer membrane proteins and in the expression of the transcriptional activator VjbR [33], [34], [35], [36]. The TCS BvrRS thereby controls the expression of *Brucella's* T4SS VirB directly (interaction between the promoter region of the VirB operon and the response regulator BvrR) and indirectly (through control of expression of VjbR by TCS BvrRS). It appears that the co-regulation of *Brucella* genes encoding the synthesis or modification of cyclic-β-1,2-glucans with those encoding the T4SS secretion apparatus and/or secreted effectors could provide additional *Brucella* factors for cyclic-β-1,2-glucan's mechanism in preventing phagosome maturation.

External stimuli such as receptor activation by microbial toxins [37], pathogens [38], [39], [40] and proteins [38], [41], [42] induce the coalescence of the small DRMDs (diameter ∼50–100 nm) that are present in resting cells into larger ones (diameter ∼500 nm to 1 μm). These clustered domains rapidly sequester proteins and lipids from detergent soluble membranes into DRMDs (within minutes of cell activation) and serve as platforms for signal transduction, intracellular sorting, membrane transport, and possibly other functions [41], [43]. We reasoned that, as seen with other pathogens [44], pathogenic *Brucella* may rearrange proteins between DRMDs and detergent soluble membranes before it enters the host cell. In this study we analyzed the protein redistribution between DRMDs and non-DRMD membranes using a quantitative proteomic approach that compares protein profiles in DRMDs of monocytes exposed or not exposed to *Brucella*.

## Materials and Methods

### Cells, bacteria and reagents

The human Mono Mac 6 cell line was obtained from the German Collection of Microorganisms and Cell Cultures (DSMZ, Braunschweig, Germany). The J774 A.1 cells were obtained from American Type Culture Collection. Mono Mac 6 cell suspensions and J774 A.1 adherent cells were cultured at 37°C under 5% CO_2_ tension in RPMI 1640 or DMEM, respectively, containing 10% FBS. For studying growth of *Brucella* in the Mano Mac 6 cells they were grown without antibiotics.

*Brucella abortus* strains RB51 (rough) and 2308 (smooth) and *B. melitensis* strains VTRM1 (rough) and 16 M (smooth) were from our bacterial collection at Virginia Tech. Before exposure of Mono Mac 6 or J774 A.1 cells, bacteria were freshly grown in Tryptic Soy Broth (TSB, BD-Difco, REF 236920) for 12 to 18 hours to ∼120 Klett Units.

The following primary mouse antibodies against human proteins were obtained from BD Biosciences: anti-flotilin-1, anti-flotilin-2, anti-transferrin receptor, anti-caveolin-1. Secondary HRP-labeled goat anti-mouse antibody was from Amersham.

Protein concentrations were determined using the BCA protein assay performed in microtiter plates (PIERCE). Cholesterol concentrations were determined using the Amplex® Red Cholesterol Assay (Invitrogen/Molecular Probes). The protease inhibitor cocktail Set III, EDTA-free was from Roche. SuperSignal West Dura Extended Duration Substrate for the development of Western Blots was from PIERCE. The iTRAQ® 4-plex labeling reagents were from Applied Biosystems. Vivaspin 2 Hydrosart Columns, 5 kDa Molecular Weight Cut-Off (MWCO) were from Sartorius Stedim Biotech. 9 kDa MWCO cellulose filtration devices were from PIERCE.

### Analysis of *Brucella* growth in Mono Mac 6 cells

Mono Mac 6 cell grown for 48 hours in suspension without gentamicin were incubated with *B. melitensis* 16 M at a MOI of 1∶100 for 1 hour at 37°C after which the cells were spun down (600×g, 9 min, 4C) and the cell culture supernatant was removed. The infected cells were washed twice with media containing 50 ug/ml gentamicin to kill all extracellular *Brucella* followed by incubation in media containing 20 ug/ml gentamicin for 0, 4, 24, 48, 72 and 96 hours. At the indicated time points infected cells were spun down at 600×g for 9 minutes and washed with media without gentamicin before the cells were lysed using 0.1% Triton X-100. Serial log dilutions of the lysates were plated on Tryptic Soy Agar (TSA) plates and incubated at 37°C for 3 to 5 days to determine the CFUs of internalized bacteria.

### Determination of time point for isolation of DRMDs

1×10^7^ Mono Mac 6 cells in suspension were exposed to rough and smooth *B. abortus* or *B. melitensis* strains at a MOI of 1∶100 for 30 seconds, 1, 4, 6, 10 or 20 minutes at 37°C in the absence of gentamicin. After the indicated incubation times, the *Brucella*-exposed cells were placed on ice. The cells were then spun down at (9 minutes, 600×g, 4°C) and washed with ice-cold media containing 50 ug/ml gentamicin to kill extracellular bacteria followed by washing with ice-cold media containing no gentamicin. Cell pellets were lysed in 0.1% Triton X-100 and serial log dilutions of the lysates were plated on Tryptic Soy Agar (TSA) plates and incubated at 37°C for 3 to 5 days to determine the CFUs of internalized bacteria.

### Confocal microscopy

Mono Mac 6 cell suspensions in media without antibiotics were exposed to freshly grown Red Fluorescent Protein (RFP)-expressing *Brucella* at a MOI of 1∶100 at 4°C for 1, 2, 4, 8, 10 and 20 minutes. Samples were centrifuged (4°C, 600×g, 9 minutes) to remove unbound *Brucella* and washed with PBS containing 50 ug/ml gentamicin. After fixing the cells in 2% paraformaldehyde they were analyzed by confocal microscopy. Transformation of *Brucella* to express RFP was performed as described previously and was found to not have an effect on invasion of host cells [45].

### Isolation of DRMDs

To isolate DRMDs 1×10^8^ unexposed Mono Mac 6 cells and cells exposed to *B. melitensis* for 2 minutes on ice (2 to 4°C) at a MOI of 1∶100 were washed with cold PBS by two cycles of centrifugation (600×g for 5 minutes). Under these conditions of exposure, all of the *Brucella* are alive and adhere to ∼75% of Mono Mac-6 cells, which have 1 to 20 *Brucella* bound to their surface.

300 ul of packed cells were re-suspended in 700 ul of ice-cold 1% Triton X-100 in HEPES-buffered saline (25 mM HEPES, 150 mM NaCl) containing protease inhibitors and passed through a 23 g needle 20 times. The resulting cell lysates were kept on ice for 10 minutes with periodic inversion of the tube. The lysates were mixed with an equal volume of 80% sucrose in HEPES buffered saline using needle and syringe to ensure that the two components are completely mixed (otherwise sucrose solutions of percentages smaller than 30% of sucrose solutions will immediately float into the upper lighter layers of the gradient). The ∼1 ml of lysates in 40% sucrose were transferred to 14×89 mm Ultra Clear™ centrifuge tubes (Beckman Coulter) and overlaid with 6.5 ml of 30% sucrose followed by 3.5 ml of 5% sucrose in HEPES-buffered saline. The samples were centrifuged in a Beckman Ultracentrifuge using the SW41 rotor at 4°C for 20 hours at 39,000 rpm (195,000×g to isopycnically separate the membranes. DRMDs by virtue of their high lipid to protein ratio are lighter than the detergent soluble membranes and float towards the top while the heavier non-detergent resistant membranes remain at the bottom. After ultracentrifugation equal volume fractions down the gradient were collected. DRMD-containing fractions were identified by their high cholesterol to protein ratio and by analyzing the presence or absence of proteins known to reside within or outside of DRMDs using Western Blotting. DRMDs were mostly contained in the fraction containing the opaque band at the interface between the 30% and 5% sucrose gradients.

### Preparation of DRMD samples for proteomic analysis

DRMD-containing fractions were pooled, solubilized in 0.5% SDS, vortexed and then filtered through Vivaspin filters. The filters were washed two times with 0.5% SDS in deionized water to remove proteins sticking to the membrane before they were reverse spun into the collection device. The Vivaspin filtration successfully removed excess lipids and sucrose from the DRMD-containing fractions. Aliquots of retentates corresponding to 100 ug protein were adjusted to equal volumes with 50 mM triethylammonium bicarbonate (TEAB), vortexed and spun down. Samples were reduced by adding 5 uL of 200 mM dithiothreitol (DTT) and heating to 56°C for 1 hour. Alkylation of cysteine residues in samples was achieved by adding 20 uL of 200 mM iodoacetamide and incubating at room temperature for 30 minutes to 1 hour in the dark. Proteins were incubated overnight with trypsin at a 1∶20 ratio of trypsin to protein (lyophilized, Promega) containing 1 mM CaCl_2_ at 37°C. The above treated samples were checked for sterility by inoculating 100 ul into TSB and incubating at 37°C for 7 days. These treated samples were found not to contain viable *Brucella* which facilitated their subsequent handling.

### Labeling of peptides with iTRAQ reagents and mass spectrometric analysis

Tryptic peptides from DRMD samples were dried using a speed-vacuum, brought up in 50 mM TEAB and then labeled with iTRAQ reagents for two hours as per manufacturer instructions. After incubation, up to four samples with the different tags were mixed and dried to remove organic solvent. The labeled peptides were cleaned by C18 spin column chromatography, dried and re-suspended in 0.1% trifluoroacetic acid (TFA). Approximately 2 ug of peptides were loaded onto the liquid chromatography column connected to a ProBot. Peptides were separated using a 85 minute gradient from 2% to 100% acetonitrile and spotted via a T-junction every 15 seconds onto MALDI plates containing Alpha cyano matrix.

Samples were analyzed by MS/MS at 1 kV using collision induced dissociation (CID) and a 4800 Plus TOF/TOF. An interpretation method was used that picked the 5 most intense peptides from the TOF analysis for fragmentation. Protein identification and quantitation analysis was performed using Protein Pilot 4.0 software (Applied Biosystems).

### Network Analysis Tool

We have developed a computational method to identify response networks in large biological networks based on expression data [46]. This method and the corresponding computer program NetworkExpress is based on superimposing expression values upon the large network, identifying *k*-shortest paths [47], [48] between seed-nodes, scoring the sub-network spanned by the set of *k*-shortest paths that are shorter than a pre-defined maximum weighted length *l*, and finding the best scored sub-network by optimization techniques. *k*-shortest paths refer to a set of paths between a start- and end-point (node) in a network with shortest, second-shortest, third-shortest connections up to a given value *k*. Paths are weighted, thus connections between intermediate nodes along the path can be shorter or longer depending on the expression values and scoring function used. We have a variety of scoring functions available, from simple arithmetic or geometric means to different types of correlation functions for time-series correlations, optionally between same time-points or time-forward/backward. The best-scored sub-network refers to the response network of the system under the specific environmental condition measured by the corresponding expression experiment.

### Gene set enrichment analysis

Our Gene Ontology (GO) enrichment analysis is based on the human annotation within the “GO Biological Process” class. The Molecular Signatures Database (MSigDB) is a collection of annotated functional gene datasets [49]. For our MSigDB enrichment analysis we used the c2.cp collection of pathway gene sets that are curated from online databases, such as BioCarta, KEGG, Pathway Interaction Database, Reactome, SigmaAldrich, Signaling Gateway, Signalt Transduction KE and SuperArray. To test for enrichment of the GO and MSigDB gene sets by iTRAQ proteins we performed Fisher Exact Tests (using a hypergeometric probability distribution) together with Benjamini & Hochberg False Discovery Rate (FDR) corrections [50]. We used 0.1 and lower as p-value cutoff.

### Network analysis

Response networks were constructed with Network Express [46] by using the corresponding iTRAQ proteins from the proteomic analysis as seed nodes from the four scenarios described in the legend for Tables 1 and 2 together with the quantitative values from the iTRAQ experiments (Table S1). Although seed nodes were restricted to the particular scenario, other proteins with complementary expression values or no expression were allowed to be present in the identified pathways if the calculated corresponding pathway scores were permissive. In other words, proteins that do not contribute to the overall pathway score during pathway calculation (i.e. proteins that do not change localization or proteins without an iTRAQ ratio) can still be visited by the Network Express algorithm during pathway calculations as long as the overall score of the particular pathway is above a pre-set minimal score (or maximal weighted length l). We used absolute log ratios as scoring function. Parameter values are k = 3 and l between 10 and 15 in the response network calculations (see section “Network Analysis Tool). Networks were drawn with Cytoscape [51]. Genome and proteome databases are updated periodically. Our initial data was analyzed using NCBI Release Version 37.1. with a release date of August 04, 2009. In order to remain consistent throughout our studies, we have frozen the genome annotation information for our functional analysis to this NCBI Release Version.

**Table 1:** Gene ontology (GO) biological processes enriched by DRMD proteins upon the exposure to the rough VTRM1 and smooth 16 M *B. melitensis* strains.

Enriched GO biological process	Contributing proteins moving into or out of DRMDs upon exposure to VTRM1	Contributing proteins moving into or out of DRMDs upon exposure to 16 M
**Ion transport**	ATP13A1*, ATP5G2*, SCN4B*, CACNA1H*	ATP13A1*, ATP5G2*, SCN4B*, SLC25A4*, VDAC2, ADD2*
**Regulation of Ras protein signal transduction**	ARFGEF1*, ARHGEF1*, USP6*	ARFGEF1, SGSM3, RAC1, TTN
**Organelle organization**	TUBB, TUBA1B, BRPF1, SPTBN4, SLC25A4, PELO, TMSL3, TTN, ADD2, KDM5C, KDM5D	
**Negative regulation of actin filament polymerization**	SPTBN4, TMSL3, ADD2	
**Cell differentiation**		CACNA1H, MYH9, TTN
**Cellular component movement**		ACTG1, RAC1, DNAH1, MYH9

Table 1 represents 84 proteins from the proteomic analysis (proteins with iTRAQ labeling) of the 149 proteins (proteins with and without iTRAQ labeling). We performed enrichment analyses based on Gene Ontology (GO) [83]. We separately analyzed rough and smooth strains as well as distinguished between proteins moving into or out of DRMDs. In both the scenarios IN and OUT we included proteins that did not change localization (0) due to the low number of iTRAQ-labeled proteins. Thus we consider four distinct scenarios: (1) VTRM1 IN + no change: 75 proteins, (2) VTRM1 OUT + no change: 50 proteins, (3) 16M IN + no change: 77 proteins, and (4) 16M OUT + no change: 49 proteins.

**Protein Key**: **ACTG1**  =  Cytoplasmic Actin 2; **ACTB**  =  cytoplasmic Actin 1; **ATP13A1**  =  Probable cation-transporting ATPase 13A1; **ATP5G2**  =  ATP synthase, mitochondrial; **ARFGEF1**  =  Brefeldin A-inhibited guanine nucleotide-exchange protein 1; **ARHGEF1**  =  Rho guanine nucleotide exchange factor 1; **ADD2**  =  Beta-adducin; **BRPF1**  =  Peregrin; **CACNA1H**  =  Voltage-dependent T-type calcium channel subunit alpha-1H; **CALM1**  =  Calmodulin; **DNAH1**  =  Dynein heavy chain 1; **KDM5C**  =  Lysine-specific demethylase 5C; **KDM5D**  =  Lysine-specific demethylase 5D; **LAMA2**  =  Laminin subunit α-2; **MYH9**  =  Myosin 9; **MYL12A**  =  Myosin regulatory light chain 12A; **PELO**  =  Protein pelota homolog; **RAC 1**  =  Ras-related C3 botulinum toxin substrate 1; **SCN4B**  =  Sodium channel beta-4 subunit; **SGSM 3**  =  Small G protein signaling modulator 3; **SLC25A4**  =  ADP/ATP translocase 1; **SPTBN4**  =  Spectrin beta chain, non-erythrocytic 4; **TTN**  =  Titin; **TMSL3**  =  Thymosin beta-4-like protein 3; **TUBB**  =  α -Tubulin; **TUBA1B**  =  α-Tubulin 1B chain; **USP6**  =  Ubiquitin carboxyl-terminal hydrolase 6; **VDAC2**  =  Voltage-dependent anion-selective channel protein 2.

Proteins marked by ^*^ did not translocate into or out of DRMDs but contributed to the enrichment of the corresponding GO biological process.

**Table 2:** MSigDB gene sets enriched by DRMD proteins upon the exposure to the rough VTRM1 and smooth 16M *B. melitensis* strains.

Enriched MSigDB gene set	Contributing proteins moving out of DRMDs upon exposure to VTRM1	Contributing proteins moving into of DRMDs upon exposure to VTRM1	Contributing proteins moving out of DRMDs upon exposure to 16M	Contributing proteins moving into of DRMDs upon exposure to 16M
**KEGG Ca^2+^ signaling pathway**	SLC25A4, VDAC2, CALM1, CACNA1H*		SLC25A4*, VDAC2*, CALM1	
**KEGG focal adhesion**	LAMA2*, MYL12A, ACTB, ACTG1		LAMA2*, MYL12A, ACTB*	
**KEGG regulation of actin cytoskeleton**	ACTB, ACTG1, ARHGEF1, MYL12A			ATCB, ACTG1, ARHGEF1*, RAC1,TMSL3*, MYH9
**REACTOME Sema4D in semaporin signaling**				MYH9, RAC1
**KEGG Gap junction/path- ogenic ** ***E. coli*** ** infection**		ACTG1*, TUBB, TUBA1B		

Table 2 represents the same proteins as described for Table 1. Proteins were analyzed in the same way as described for Table 1 with the exception of the enrichment analysis, which is based on MSigDB gene sets instead of GO (Table 1) [83]. Proteins marked by ^*^ did not translocate into or out of DRMDs but contributed to the enrichment of the corresponding MSigDB gene set. The protein key is also the same as the one provided for Table 1.

## Results, Discussion & Conclusions

### Growth of *Brucella* in Mono Mac 6 cells

**Figure 1:**
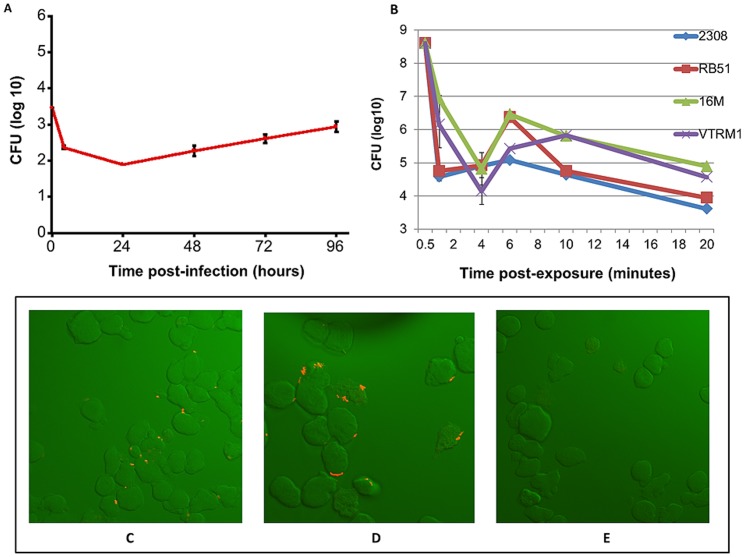
Growth of *Brucella* in Mono Mac 6 cells and attachment of *Brucella melitensis* to Mono Mac 6 cells. **Figure 1** **A** shows the survival of *B. melitensis* 16 M in Mono Mac 6 cells over 96 hours at 37°C. **Figure 1** **B** depicts the internalization of rough and smooth *B. melitensis and B. abortus* strains into Mono Mac 6 cells at 37°C for up to 20 minutes. *B. abortus* strains appear to enter Mono Mac 6 cells slightly faster (within about 1 minute) than *B. melitensis* strains (within about 4 minutes) as indicated by the lowest CFU numbers which represent the time when all bacteria have become intracellular (extracellular bacteria have been killed by gentamicin and intracellular bacteria are not multiplying yet). The confocal images in **Figures 1** **C and D** show the adherence of RFP-expressing *B. melitensis* to Mono Mac 6 cells at 1 minute (**C**) and 2 minutes (**D**) upon exposure at 4°C. Mono Mac 6 cells not exposed to *Brucella* are shown in **Figure 1** **E**.

Many studies addressing the uptake of *Brucella* by macrophages and epithelial host cells have been conducted, but virtually none with Mono Mac 6 cells [52] [53]. Since we had not performed such experiments in our laboratory, we set out to analyse the invasion and growth of *Brucella* into/in Mono Mac 6 cells. The growth curve in Figure 1 A shows that smooth *B. melitensis* 16M is efficiently taken up by Mono Mac 6 cells. Based on our experience with *B. abortus*, *B. suis* and *B. melitensis* in J774 A.1 mouse macrophages and at a MOI of 1∶100 it appears that the Mono Mac 6 cells are more bacteriocidal by about 1 to 2 logs over the first 4 hours. However, within the first 24 to 48 hours the surviving *Brucella* grow back to infection levels observed in the beginning. These results demonstrate that that the characteristics of Mono Mac 6 cells are well suited for studying cellular responses induced by the exposure to *Brucella* [52], [53].

### Determination of time point for extraction of DRMDs

Previous studies suggested that initiation of cell signaling leading to Brucella invasion are linked to changes in the protein composition of DRMDs in the host cell plasma membrane which start at the time host cells come in contact with *Brucella* [8], [11]. In order to capture these early changes we needed to isolate DRMDs before *Brucella* invade Mono Mac 6 cells.

To determine the time it takes for *Brucella* to enter Mono Mac 6 cells we exposed Mono Mac 6 cells to the rough and smooth *Brucella melitensis* and *Brucella abortus* strains for 0.5, 1, 4, 6, 10 and 20 minutes at 37°C followed by immediate cooling to 4°C (Figure 1 B). The time points at which the lowest CFU numbers are observed represent the time when all bacteria have become intracellular (extracellular bacteria have been killed by gentamicin and intracellular bacteria are not multiplying yet). The high CFU numbers of about 5×1×10^8^ at the 30 second time point for all *Brucella* strains seem to indicate that gentamicin did not have enough time to be effective in killing all of the extracellular *Brucella*. *B. abortus* strains appear to enter Mono Mac 6 cells slightly faster (within about 1 minute) than *B. melitensis* strains (within about 4 minutes). Even though the Mono Mac 6 cells were immediately cooled on ice upon incubation with Brucella for 0.5, 1, 2, 10 and 20 minutes, the actual incubation times at temperatures above 4°C are somewhat longer because it takes additional time to bring the temperature of the cell suspensions down to 4°C. The prolonged decreasing slopes for *B. melitensis* compared to *B. abortus* (Fig. 1 B) could indicate an increased resistance to killing of extracellullar *B. melitensis* by gentamicin. This is supported by the higher CFUs for *B. melitensis* at the 1 minute time point and the fact that there is little difference in the lowest CFUs observed for *B. abortus* and *B. melitensis*.

We find no difference in the kinetics of entering Mono Mac 6 cells between the rough and smooth strain of *B. abortus* (RB51 and 2308, respectively) and the rough and smooth strain of *B. melitensis* (VTRM1 and 16M, respectively). The similar patterns for internalization of the smooth (2308 and 16M) and rough (RB51 and VTRM1) *B. abortus* and *B. melitensis* strains into Mono Mac 6 cells suggest that these smooth and rough strains are internalized by the same or a similar pathway. This is in contrast to a previous study that showed that the internalization of the non-opsonized smooth S2308 *B. abortus* strain into J774 A.1 cells occurs much faster (∼3 min) than that of the rough CA180  =  S2308 *man*BA::Tn5 = CA180 strain (>35 min) [54]. The respective authors concluded that this was due to the absence of a complete *Brucella* O-polysaccharide in the LPS of the rough strain, which restricts its overall binding and uptake. While there might be other factors that contribute to our finding a previous characterization of the LPS expressed by *B. abortus* RB51 shows that this naturally occurring rough mutant produces low levels of M-like O-antigen, a typical smooth-lipopolysaccharide (S-**LPS**) pattern which resembled that of M-dominant S-**LPS of** *B. melitensis* [55]. Whether VTRM1 also produces low levels of a S-LPS is currently unknown. Because of the increased pathogenicity of *B. melitensis* compared to *B. abortus* we performed our forthcoming proteomic studies with this species.

Because of the short time (1 to 4 minutes) it takes *Brucella* to invade Mono Mac 6 cells at 37°C and our intent to capture changes to DRMDs in Mono Mac 6 cells before *Brucella* entry we exposed *Brucella* to Mono Mac 6 for the lipid raft extractions at 4°C. We performed confocal microscopy studies of RFP-expressing *B*. melitensis (Figures 1 C & D and Video S1) to confirm that *B. melitensis* adheres to Mono Mac 6 cells at 4°C. We previously determined that the expression of the red fluorescent protein in *B. melitensis* has no effect on the invasion properties of *Brucella* (Sriranganathan, unpublished). Figures 1 C & D are representative images of more than 20 stacked sections taken through the depth of the cell by confocal microscopy. They show that *B. melitenesis* adheres to the plasma membrane of Mono Mac 6 cells at 1 and 2 minutes after exposure. To confirm that the bacteria have not entered the Mono Mac 6 cells under these conditions we analyzed reconstructions of the individual sections. The Video S1 was reconstructed from 27 individual stack images taken through the depth of a Mono Mac 6 cell and confirms that 4 minutes after their exposure to *B*. *melitensis* the bacteria have attached to, but not entered Mono Mac 6 cells (Video S1 for M16 strain, data not shown for VTRM1). Please note that the red dots in the upper half of the cells that come up in the second part of the video are out of focus and result from an adherent cell. We therefore chose to extract DRMDs from Mono Mac 6 cells two minutes after exposure to *B. melitensis* at 4°C since under these conditions B. *melitensis* does not enter Mono Mac 6 cells (Figure 1 C through E, Video S1).

### Comparative proteomic analysis of DRMDs from unexposed and *Brucella*-exposed cells using iTRAQ labeling

**Figure 2:**
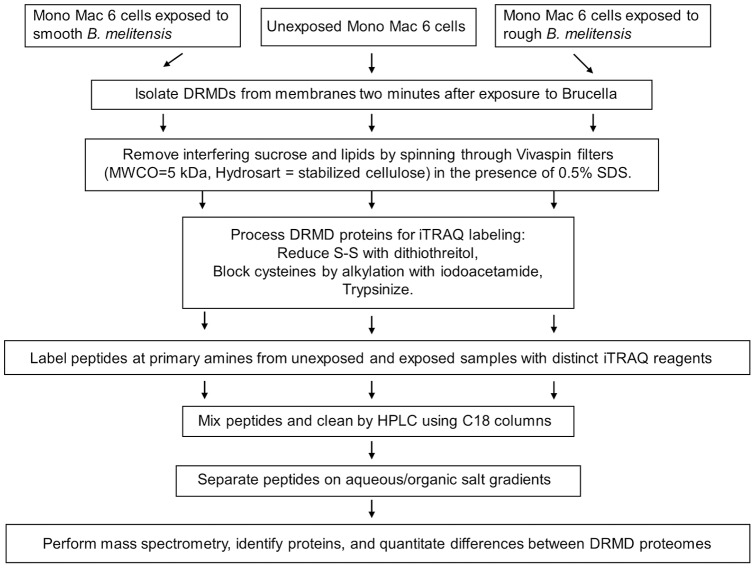
Processing of DRMDs for the comparative proteomic analysis using the iTRAQ method. The schematic shows the work flow for processing of DRMD samples from uninfected and infected cells for the quantitative mass spectrometry analysis using the iTRAQ method. Interfering lipids and sucrose were removed using Vivaspin filters before DRMD samples were reduced with dithiothreitol, followed by alkylation with iodoacetamide and trypsinization. These treatments are routinely used in the preparation of samples for labeling with iTRAQ reagents for the mass spectrometric analysis were found to effectively kill *Brucella*.

DRMDs for the iTRAQ labeling and subsequent mass spectrometric analysis were isolated from unexposed Mono Mac 6 cells and Mono Mac 6 cells exposed for 2 minutes to *B. melitensis* 16M (smooth strain) and VTRM1 (rough strain) (Figure 2). Under these conditions (2 minute exposure at 2 to 4°C at a MOI of 1:100) all of the *Brucella* are alive and adhere to ∼75% of Mono Mac-6 cells, which have 1 to 20 *Brucella* bound to their surface. Even though the live *Brucella* are in physical contact with host cells at the 2 minute time point, we cannot rule out that some of the observed results are caused by diffusible products produced by *Brucella*.

Because of the short incubation period of two minutes metabolic labeling of proteins could not be performed. We therefore chose to label proteins in DRMDs using iTRAQ labeling of the protein-derived peptides (Figure 2). Many proteomic studies rely on single mass spectrometric datasets, which increases the probability of incorrect reporting of proteins. We performed three independent extractions of DRMDs from unexposed and *Brucella*-exposed Mono Mac 6 cells and determined the average iTRAQ ratios (see Table S1, Columns C and D). The iTRAQ ratio represents the amount of a particular protein in DRMDs of Mono Mac 6 cells exposed to *Brucella* against the background of unexposed Mono Mac 6 cells.

We observed 149 proteins (Table S1) in DRMDs upon *Brucella* exposure at least two times out of the three independent experiments. About 80 additional proteins were found only once in DRMDs upon *Brucella* exposure (data not shown). These proteins were neglected in our subsequent analyses. Among the 149 proteins were 29 signaling proteins, 22 structural/cytoskeleton/motor proteins, 17 proteins involved in transcriptional regulation, 17 transport proteins, 8 adhesion proteins, and 27 proteins of currently unknown function (Table S1).

In columns C and D of Table S1 we report the averaged iTRAQ ratios and their standard deviations (SD) for the rough VTRM1 and the smooth 16M *B. melitensis* strain, respectively. An iTRAQ ratio of “1” means that the amount of a particular protein in DRMDs is the same in unexposed and *Brucella*-exposed Mono Mac 6 cells. A ratio above “1” means that a particular protein is more abundant in DRMDs of the *Brucella*-exposed sample, a ratio below “1” indicates that a protein is less abundant in DRMDs of the exposed sample. The Extreme High Value (EHV) corresponds to a ratio of  =  or >100. The Extreme Low Value (ELV) corresponds to a ratio of  =  or <0.01.

In columns E and F of Table S1 we report the iTRAQ ratios semi-quantitatively in terms of the extent of translocation of a protein into (+, ++, +++, ++++) or out (-, —, ---, ----) of DRMDs upon *Brucella* exposure, or the lack thereof (0). We based our quantification on typical use of iTRAQ labeling in which we set a 25% threshold for the iTRAQ ratio above or below 1 to signal the translocation of a protein into or out of DRMDs upon *Brucella* exposure [56]. Therefore, proteins with iTRAQ ratios between 0.75 and 1.25 are considered to have not changed their location upon exposure, which is denoted as “0”. An increase in the iTRAQ ratio above 1.25, denoted by one or more plus signs, indicates that a protein moved from detergent soluble membranes into DRMDs upon *Brucella* exposure. Similarily, a decrease in the ratio below 0.75, denoted by one or more minus signs, indicates that a protein moved from DRMDs into detergent soluble membranes upon exposure. We further arbitrarily quantified the extend of translocation of proteins into and out of DRMDs by assigning the following ranges and semiquantitative values for the iTRAQ ratios: Range 1.25 to 2 = “+”; Range >2 to 10 =  “++”; Range >10 to <100 =  “+++”; Extreme High Value (EHV)  = /> 100 =  “++++”; Range 0.75 to 0.50 =  “-”; Range <0.5 to 0.25 =  “—”; Range <0.25 to >0.01 =  “---”; Extreme Low Value (ELV)  = /<0.01 =  “----”.

### Identification of proteins that re-distribute between DRMDs and detergent soluble membranes due to *Brucella* exposure

Since DRMDs function as signaling platforms, host cell proteins that move between DRMDs and non-DRMD membranes upon exposure to *Brucella* are expected to play a role for invasion and/or subsequent steps of the infectious process. Differences in the extent of movement of such proteins due to the exposure to rough and smooth *Brucella* strains could thus provide indicators for virulence.

We find that 24 proteins moved into DRMDs due to the exposure to either the rough VTRM1 or the smooth 16M strain (Table S1). Accumulation for 22 of these proteins was similar for the smooth and rough strain (Table S1). The increase of the small G protein signaling modulator 3 was much higher upon exposure to the rough VTRM1 strain compared to the smooth 16M strain (Table S1). In addition, the small G protein signaling modulator 3 (SGSM3) was found to enrich the regulation of Ras protein signal transduction upon exposure to the smooth strain (Table 1).

The small G protein signaling modulator 3 has been shown to co-precipitate with several of the small GTPases and to positively regulate Rab GTPase activities, which function in signal transduction and vesicular trafficking pathways [57]. Trafficking of the BCV along the endocytic and secretory pathways is controlled by the bacterium. During maturation along the endocytic pathway, the BCV becomes acidified and acquires late endosomal markers, before it is redirected towards the early secretory pathway through intimate interactions with the endoplasmic reticulum exit sites (ERES). The BCV eventually fuses with the ER in a process that depends upon the small GTPase Sar1 and thus on the formation of COPII-dependent transport vesicles. During this process, the BCV recruits the small GTPase Rab2 and GAPDH that regulate membrane traffic between the ER to Golgi intermediate compartment (ERGIC), and are required for *Brucella* replication [58]. Our results suggest that the small G protein signaling modulator 3 could play a role in the differential regulation of Rab2-dependent pathways that lead to the replication of the smooth, but not the rough *Brucella* strain.

For thymosin beta 4-like protein the accumulation in DRMDs due to the exposure to the smooth strain was somewhat higher compared to the rough strain. Thymosin beta 4-like protein binds to and sequesters actin monomers (G actin) and thereby inhibits actin polymerization [59]. However, we found large standard deviations associated with the iTRAQ values for this protein. Currently, there is no notion that alterations in thymosin beta-4 like protein are linked to *Brucella's* interference with actin polymerization.

11 proteins moved into DRMDs due to exposure to the rough strain, but no change was observed when the cells were exposed to the smooth strain (Table S1). 15 proteins moved into DRMDs due to exposure to the smooth strain, but no change was observed when the cells were exposed to the rough strain (Table S1). For one of these proteins, myosin 9, the amount was extremely high upon exposure to the smooth strain. Myosin 9 enriches the GO functions cell differentiation and cellular component movement (Table 1) and the MSigDB gene sets KEGG regulation of actin cytoskeleton and REACTOME Sema4D in semaporin signaling (Table 2) upon exposure to the smooth M16 strain only.

Myosin 9 is a non-muscular myosin IIA that appears to play a role in cytokinesis, cell shape, and specialized functions such as secretion and capping. Recently, a non-muscular myosin IIA (NMM-IIA) in J774A.1 macrophages was found to serve as a receptor or an adaptor protein (necessary to engage the functional receptor) for the *Brucella* virulence factor PrpA [60]. PrpA-treated macrophages induced the secretion of a soluble factor responsible for B-cell proliferation and establishment of the chronic phase of the infectious process. The NMM-IIA identified by Spera et al. has a molecular weight of approximately 225 kDa. Myosin 9 has a reported molecular weight of 226 kDa. Since the exposure to the smooth strain triggered an accumulation of myosin 9 in DRMDs, which are known to serve as signaling platforms, it is very likely that the myosin 9 identified by us could be the NMM-IIA receptor or adaptor identified by Spera et al. or could serve its function in PrpA virulence. It would be interesting to explore whether PrpA exposure triggers myosin 9 accumulation in DRMDs of Mono Mac 6 cells and whether neutralization of myosin 9 in Mono Mac 6 cells abolishes the binding of PrpA and B-cell proliferation. The increased accumulation of myosin 9 in DRMDs upon exposure to the smooth, but not the rough strain, is very consistent with the smooth strain's ability to successfully establish chronic infections in the host and the inability of the rough strain to do so.

28S ribosomal protein S35, a component of the mitochondrial ribosome small subunit (28S) which comprises a 12S rRNA and about 30 distinct proteins, moved into DRMDs upon exposure to the rough strain, but was severely depleted from DRMDs upon exposure to the smooth strain. This protein has been shown to be involved in the response to DNA damage and protein synthesis within the mitochondrion [61]. Whether and how the 28S ribosomal protein S35 might be involved in the differential response to rough and smooth *Brucella* remains currently unknown.

Eight proteins moved out of DRMDs due to the exposure to the rough and smooth strains. For four of these proteins the amounts detected in DRMDs upon exposure to the rough and smooth *Brucella* strains were similar. For the disintegrin and metalloproteinase with thrombospondin motifs 18 preproprotein and the large subunit GTPase 1 homolog the amounts detected in DRMDs after exposure to rough and smooth strains were somewhat lower for the rough strain. For lysine-specific demethylase 5D and erlin-2, the amounts detected in DRMDs after exposure to the rough strain were much lower than those detected in DRMDs upon exposure to the smooth strain.

The disintegrin and metalloproteinase with thrombospondin motifs 18 functions in protein degradation. There is accumulating evidence that such proteinases play various roles in bacterial and viral infections. The effects of such proteinases vary with the specific protease, infectious agent, and type of host cell or tissue and range from host tissue destruction in *H. plyori*, *M. tubercolosis* and HIV infections to disruption of bacterial membranes in *S. aureus* [62]. At this time the role of this proteinase for infection with rough and smooth *Brucella* remains to be discovered.

The large subunit GTPase 1 homolog appears to be involved in the release of NMD3 from the 60S ribosomal subunit after export into the cytoplasm [63]. Whether and how this protein might be involved in the differential response to rough and smooth *Brucella* is currently unknown.

Lysine-specific demethylases play central roles in the histone code [64], [65]. This suggests that the exposure of Mono Mac 6 cells to rough and smooth *Brucella* somehow differentially alters its transcriptional regulation due to histone protein modifications. We find that the lysine-specific demethylases 5D (KDM5D) and 5C (KDM5C) enrich the GO function organelle organization upon the exposure of Mono Mac 6 cells to the rough VTRM1 strain (Table 1). It remains to be discovered how the changes to organelle organization induced by the exposure to *Brucella* are linked to changes in transcription due to histone code alterations.

Erlin-2, a component of the Erlin 1/Erlin 2 complex, which mediates the endoplasmic reticulum (ER)-associated degradation of inositol 1,4,5-trisphosphate receptors, is an ubiquitylation-dependent elimination pathway of misfolded proteins in the endoplasmic reticulum [66], [67], [68]. Smooth *Brucella* replicate within a BCV that contains ER resident proteins while rough *Brucella* do not. Currently, the molecular mechanisms by which *Brucella* regulate intracellular trafficking and replication to exploit this intracellular niche are largely unknown. In a RNAi screen for molecules that mediate *Brucella* interactions with the ER Quin et al. uncovered 52 evolutionarily conserved host factors that inhibited or increased *Brucella* infection upon depletion and proposed a model in which ER-associated genes may mediate *Brucella* replication by promoting autophagosome biogenesis [69]. The model suggests that the accumulation of BCVs in the ER activates IRE1α, which in the following may trigger the biogenesis of ER-containing autophagosomes. Activation of the ER stress sensor IRE1α depends on its direct interaction with unfolded proteins and is induced by changes in membrane composition which then lead to the initiation of various signaling pathways that regulate genes involved in ER protein synthesis, folding, glycosylation, ERAD, redox metabolism, autophagy, lipid biogenesis and vesicular trafficking [70]. Indeed, it has recently been shown that smooth *B. melitenis* provokes an unfolded protein response[71]. Our results suggest that the severe downregulation of Erlin-2 in DRMDs upon exposure to the rough strain is somehow inhibits requirements for its replication in an ER-like compartment such as autophagosome biogenesis and unfolded protein responses.

Eight proteins moved out of DRMDs due to the exposure to the rough strain, but no change was observed upon exposure to the smooth strain. One of these proteins, voltage-dependent anion-selective channel protein 2 was severely depleted in DRMDs upon exposure to the rough strain. Voltage-dependent anion-selective channel protein 2 is an outer mitochondrial membrane protein that forms a channel through the mitochondrial outer membrane and allows diffusion of small hydrophilic molecules. This protein has been implicated in the negative regulation of intrinsic apoptotic signaling pathways and in the negative regulation of protein polymerization [72], [73]. We find that Voltage-dependent anion-selective channel protein 2 enriches the ion transport GO function upon the exposure to the smooth strain only (Table 1) and the MSigDB gene set KEGG Ca^2+^ signaling pathway upon exposure to the rough and smooth *Brucella* strains (Table 2).

He's group reported that the rough attenuated *Brucella* strains such as *B. abortus* cattle vaccine strain RB51 and *B. suis* vaccine candidate VTRS1 induce a caspase-2-mediated, caspase-1-independent pro-inflammatory cell death in infected macrophages and dendritic cells (“caspase-2-mediated pyroptosis”) which is driven by mitochondrial dysfunction [74], [75], [76], [77]. Interestingly, smooth and virulent *Brucella* inhibit such cell death in infected macrophages [78], but not in dendritic cells [77]. We currently do not understand whether the severe depletion of the voltage-dependent anion-selective channel protein 2 in DRMDs upon exposure to the rough *B. melitensis* strain contributes to mitochondrial dysfunction that leads to the caspase-2-mediated pyroptosis observed by He and coworkers. However, the depletion of this anti-apoptotic protein from DRMDs upon exposure to the rough strain occurs right upon exposure of Mono Mac 6 cells to *Brucella*.

Four proteins moved out of DRMDs due to the exposure to the smooth strain, but no change was observed upon exposure to the rough strain. For three of these four proteins, nuclear autoantigen Sp-100, WD repeat-containing protein 31 and zinc finger protein 746, the amounts in DRMDs were very low upon exposure to the smooth strain.

The nuclear autoantigen Sp-100 is a transcriptional regulator and a major constituent of nuclear bodies, a sub-nuclear organelle involved in a large number of physiological processes including cell growth, differentiation and apoptosis [79]. It also has been shown to play a role in infection by viruses (human cytomegalovirus and Epstein-Barr virus) through mechanisms that may involve chromatin and/or transcriptional regulation [80], [81]. The role this protein plays for infections with rough and smooth *Brucella* is currently unknown.

The function of WD repeat-containing protein 31 is currently not known.

Zinc finger protein 746 appears to specifically bind to the 5′-TATTTT[T/G]-3′ consensus sequence on promoters to repress transcription and thereby plays a role in regulation of cell death [82]. Whether and how this protein might be involved in the differential response to rough and smooth *Brucella* remains to be uncovered.

For 16 proteins we did not observe movement between DRMDs and non-DRMD membranes upon exposure to the rough VTRM1 and smooth *B. melitensis* strains. Seven proteins moved into DRMDs upon exposure to the rough strain, but we failed to label peptides in samples that were exposed to the smooth strain (Table S1). Another six proteins moved into DRMDs due to the exposure to the smooth strain, but we were unable to label peptides in samples that were exposed to the rough strain (Table S1). An additional 51 proteins were detected in DRMDs upon exposure to the rough and smooth strain, but the labeling efficacy was insufficient to assign iTRAQ values.

Taken together, we identified a number of host proteins that display differences in the iTRAQ ratios upon exposure to the rough and smooth *Brucella* strains and discussed our findings based on current knowledge in *Brucella*-host interactions. For some of these proteins we found extreme high or low iTRAQ ratios (i.e. EHV and ELV in Table S1 for myosin 9, small G protein signaling modulator 3, lysine-specific demethylase 5D, erlin-2, 28S ribosomal protein S35, voltage-dependent anion-selective channel protein 2, nuclear autoantigen Sp-100, WD repeat-containing protein 31 and zinc finger protein 746). The verification of these findings by independent methods is outstanding. Furthermore, the foregoing analysis of individual proteins does not allow us to deduce information of cellular pathways and networks that might be affected by the exposure/adherence to/of *Brucella*. In order to understand how *Brucella* exposure/adherence alters cellular pathways and networks in Mono Mac 6 we used the identified proteins to perform the analyses described below.

### Enrichment analysis and identification of cellular pathways and networks that change in response to the exposure of Mono Mac 6 cells to *B. melitensis*

Cellular functions and pathways depend on sets of proteins acting in concert. Increases or decreases in the expression of proteins constituting a pathway can have striking effects which may be more important than a particular large increase or decrease of a single protein. In order to elucidate cellular pathways and networks that might be affected by cellular insults such as the exposure to *Brucella* the expression of sets of proteins within pathways needs to be analyzed. We therefore determined whether DRMD-based proteins (Table S1) concordantly enrich Gene Ontology (GO) biological functions (Table 1) and MSigDB gene sets (Table 2) in Mono Mac 6 cells.

We performed an analysis based on BiNGO [83] to identify GO biological functions that are enriched upon exposure to the rough and smooth *B. melitensis* strains. GO biological functions enriched upon exposure to the rough and smooth *Brucella* strains and associated DRMD proteins are presented in Table 1. We find that ion transport and regulation of Ras protein signal transduction are enriched in Mono Mac 6 cells upon exposure to both the smooth as well as the rough *Brucella* strain. The proteins that contribute to the enrichment of these GO processes common to the rough and smooth strain are mostly different, but some are the same. Proteins that did not change localization (0) were included in both the scenarios IN and OUT due to the low number of iTRAQ-labeled proteins and are marked by *. Additional GO processes enriched upon exposure to the rough strain are organelle organization and negative regulation of actin filament polymerization. GO processes enriched upon exposure to the smooth strain are cell differentiation and cellular component movement (Table 1).

Using the same iTRAQ-labeled proteins as for the GO analysis and conditional constraints we identified the following enriched MSigDB gene sets. KEGG Calcium Signaling Pathway and KEGG Focal Adhesion are commonly enriched by proteins moving out of DRMDs due to the exposure to the rough VTRM1 and smooth 16M strain. The proteins enriching the KEGG Calcium Signaling Pathway (SLC25A4, VDAC2, CALM1) and KEGG Focal Adhesion (LAMA2, ACTB, MYL12A) are mostly the same, but there are additional proteins that contribute to the enrichment of these two gene sets when cells are exposed to the rough strain.

KEGG Regulation of Actin Cytoskeleton is enriched by proteins moving out of DRMDs due to the exposure to the rough VTRM1 strain and by proteins moving into DRMDs due to the exposure to the smooth 16M strain. ATCB, ACTG1, ARHGEF1 commonly contribute to the enrichment of KEGG Regulation of Actin Cytoskeleton, but in opposite ways (i.e. by moving out of DRMDs upon exposure to the rough strain versus moving into DRMDs upon exposure to the smooth strain). There are also additional proteins that contribute to the enrichment of these two gene sets upon exposure to the rough and smooth strain.

Individually enriched pathways are the Reactome Sema4D in Semaporin Signaling and KEGG Gap Junction/Pathogenic *Escherichia coli* Infection. The Reactome Sema4D in Semaporin Signaling pathway is specifically enriched by MYH9 and RAC1 which move into DRMDs upon exposure to the smooth 16M strain only. The KEGG Gap Junction/Pathogenic *Escherichia coli* Infection pathway is specifically enriched by ACTG1, TUBB and TUBA1 upon exposure to the rough VTRM1 strain only.

**Figure 3:**
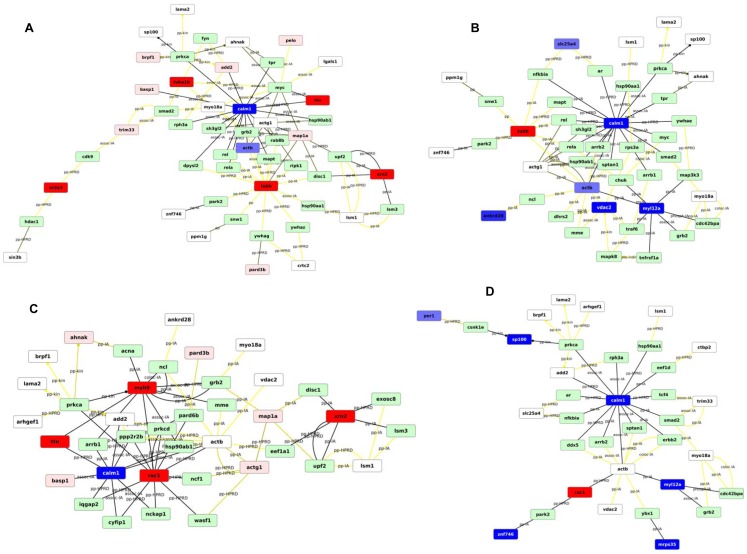
Networks of proteins based on enriched gene ontology functions. Response networks were constructed with Network Express [46] by using the corresponding iTRAQ proteins as seed nodes from the four scenarios described in the legend for Tables 1 and 2 together with the quantitative values from the iTRAQ experiments. Networks were drawn with Cytoscape [51].**A**: VTRM1 IN + no change; **B**: VTRM1 OUT + no change; **C**: 16M IN + no change, **D**: 16M OUT + no change. IN  =  +, ++, +++ or ++++. OUT  =  -, —, --- or ----. **Nodes**: red: up-regulated (IN) blue: down-regulated (OUT) white: no change (0) green: no data. The more red/blue, the higher the fold-change. Full red/blue for the highest bracket of the iTRAQ scores. **Edges**: black: high score (“strong/likely connection”) yellow: low score (“weak/less likely connection”).

We further performed an analysis based on the enriched GO functions to identify cellular response networks that become activated due to the exposure to the rough and smooth *B. melitensis* strain (Figure 3). We find that exposure to the rough VTRM1 strain induces apoptotic pathways and cell death, affects the regulation of the NFkB pathway, cell development, the response to chemical stimuli, and IL12 production, while exposure to the smooth strain affects networks regulating the cytoskeleton and signaling pathways (Figure 3).

Our results show that the rough and smooth *B. melitensis* strains induce distinct DRMD-associated pathways and response networks in Mono Mac 6 cells immediately upon *Brucella* encounter. The rough VTRM1 strain induces cytotoxic effects on Mono Mac 6 cells while the smooth *B. melitensis* 16M strain stimulates cytoskeletal changes and signaling pathways. Previous studies have shown that rough *Brucella* strains are cytotoxic to host macrophages by inducing pyroptotic cell death [75], [78], [84], [85] and that the entry of *Brucella* into host cells depends on cytoskeletal rearrangements and associated signaling pathways [86], [87], [88]. However, it had not been shown that these pathways involve rearrangements of proteins between DRMDs and non-DRMD membranes and that these occur immediately upon the encounter of *Brucella*. Thus, *Brucella* exposure to MonoMac 6 cells appears to facilitate the invasion of the rough and smooth strains, as well as subsequent downstream events leading to pyroptotic macrophage death by the rough strain and inhibition of macrophage death by the smooth strain [89], [90], [91], [92].

## Supporting Information

Video S1 **RFP-expressing** ***B. melitensis*** **adhering to Mono Mac 6 cells.** The video was constructed from 27 individual sections that were taken through the depth of a Mono Mac 6 cell by confocal microscopy and shows that *B. melitensis* adheres to the outside of Mono Mac 6 cells but has not entered the cells 4 minutes after exposure.
(AVI)

Table S1 **Human proteins identified by mass spectrometry that change their association with DRMDs upon exposure with** ***B. melitensis*** **.** Mass spectrometric data was analyzed with ProteinPilot Software 4.0 and iTRAQ ratios were calculated. Three separate experiments were performed. Proteins, which were observed only one time in the three experiments were ignored and are not shown. Proteins, which were observed at least two times out of the three experiments are shown. For these proteins we report the average iTRAQ ratios and their standard deviations (SD, Columns C & D). ProteinPilot sets the upper and lower limits of the iTRAQ ratios at 100-fold and arbitrarily reports ratios of  = />100 as extreme high values (EHV) and ratios of  = /<0.01 as extreme low values (ELV). Since the extreme values of 100 and 0.01 are set arbitrarily their standard deviations cannot be obtained. Some of the proteins were observed at least two times but had iTRAQ ratios associated with them in only one experiment. For these proteins no standard deviation is associated with their iTRAQ ratio. Other proteins were observed at least two times but we failed to label them in every experiment. These proteins are identified by “no iTRAQ#”. For Columns E & F the following set of rules were applied: In accordance with typical use of iTRAQ labeling [56], if the iTRAQ ratio for a particular protein did not change more than 25% upon *Brucella* exposure, it was considered to not have changed and is denoted “0”. “No data” means that we failed to observe iTRAQ labeling, but found the protein to be present in DRMDs. If the iTRAQ ratio was at least 25% below 1 (i.e. <0.75) the protein was considered to have moved out of DRMDs upon *Brucella* exposure and is denoted by a negative sign. If the iTRAQ ratio was at least 25% above 1 (i.e. >1.25) the protein was considered to have moved into DRMDs upon *Brucella* exposure and is denoted by a positive sign. In order to distinguish the extend of movement of a protein into or out of DRMDs we assigned the following arbitrary ranges and semiquantitative values: Range 1.25 to 2 = “+”; Range >2 to 10 = “++”; Range >10 to <100 =  “+++”; Ratio of EHV  = 100 =  “++++”; Range 0.75 to 0.50 = “-”; Range <0.5 to 0.25 =  “—”; Range <0.25 to >0.01 = “---”; Ratio of ELV  = 0.01 =  “----”.
(XLSX)
